# Diagnostic value of an ROC curve of the size of the antepartum foramen ovale in the prediction of puerperal atrial septal defect

**DOI:** 10.3892/etm.2013.1012

**Published:** 2013-03-15

**Authors:** LIN LIU, YI-HUA HE, ZHI-AN LI, YE ZHANG, XIAO-YAN GU, JIAN-CHENG HAN, JIAO-YANG CHEN

**Affiliations:** 1 Department of Medical Ultrasound, Henan Provincial People’s Hospital, Zhengzhou, Henan 450003;; 2 Department of Medical Ultrasound, The Affiliated Beijing Anzhen Hospital of The Capital Medical University, Beijing 100029, P.R. China

**Keywords:** foramen ovale, atrial septal defect, antepartum diagnosis, ROC curve

## Abstract

The aim of this study was to determine the diagnostic value of an ROC curve of the antepartum foramen ovale (AFO) size and the ratio of FO size to aorta (AO) size (FO/AO) for the prediction of puerperal atrial septal defect in different gestational weeks (DGWs). A total of 958 cases were divided into five groups according to number of gestational weeks. Comparisons of FO size, AO size and FO/AO were determined by variance analysis. The correlations between FO size, AO size and gestational age were determined using regression analysis and comparisons between atrial septal defect (ASD) diagnosed in DGWs and normal cardiac FO size and FO/AO were analyzed by t-test. ROC curve analysis was used for FO size and FO/AO to predict the demarcation point of puerperal ASD (pASD). The differences between FO size and AO size in the five groups at DGWs were statistically significant (P=0.000). The sizes of FO and AO increased with gestational age. The differences among pASD, normal cardiac FO size and FO/AO were statistically significant (P=0.000). FO size in the five DGW groups (18–22, 23–26, 27–30, 31–34 and 35–40 weeks) was able to predict the demarcation points of pASD, which were 5.02, 5.15, 6.55, 8.55 and 7.90 mm, respectively. The prediction of pASD with AFO size and FO/AO was accurate and may provide reliable reference values in the clinic.

## Introduction

The foramen ovale (FO) is one of the special channels in the fetal period, which shunts blood reflowing from the vena cava to the left cardiac system to allow regular circulation. It is open prenatally, then it closes puerperally with the increasing pressure of the left heart, which forces the FO valve to move forward to the septum secundum (1–5). When the size of the FO becomes excessively large, it produces a foramen secundum atrial septal defect (ASD). This study discusses the clinical value of FO size and the ratio of FO and aorta size (FO/AO) in ROC curve analysis for the prediction of pASD.

## Subjects and methods

### Subjects

A total of 1,690 cases diagnosed using fetal ultrasonic cardiogram (FUC) from September 2009 to March 2012 were selected. The inclusion criteria were that the relative prenatal and puerperal FUC data were accessible and the diagnosis of the cardiogram was normal. The exclusion criteria were cardiac abnormalities in the ultrasonic cardiograms, ratio imbalance of the dextrocardia and levocardia and incomplete data for follow-up. Informed consent was provided by the subjects themselves or their relatives.

### Instruments

FUC was performed using a GE Voluson E8 diasonogram with an RAB4-8 probe at a frequency of 4–8 MHz in the ‘fetal cardiac’ mode. Puerperal re-examination of ultrasonic cardiogram (PUC) was performed using a Philips iE33 ultrasound apparatus with a S5-1 probe at a frequency of 1–5 MHz in the ‘paediatrics’ mode (Philips Ultrasound, Inc., Bothell, WA, USA).

### Routine examination

The first examinations were biological indicator investigations, including biparietal diameter (BPD), head circumference, abdominal perimeter and femur length (FL) to verify the fetal gestational weeks. Then FUC examinations were performed according to the standard guide of FUC recommended by the United States Association of Echocardiography (6). The scanning sequences of the standard plane were: abdominal cross section, four chamber view, left ventricular outflow tract (LVOT) section, right ventricular outflow tract (RVOT) section, double ventricular short axis view, three vessels and trachea section, long axis view of the aortic arch and long axis view of the *ductus arteriosus*. Cardiac abnormalities were excluded according to the above section screening.

### Measurement of FO and AO size

The best section for FO measurement was from the transverse four chamber view. When the valve of the FO was observed clearly and was opened extensively, the measurement was performed under the condition of two dimensional ultrasound, three times for the determination of the average value. The inner diameter of the AO root was measured at the LVOT section in systole by processing three times for the mean value.

### Puerperal re-examination

FUC was rechecked no less than 12 months after parturition, in accordance with the standard section, including the left ventricular long axis view, short axis section of the large artery, short axis section of the two ventricles, four chamber view, five chamber view, sword double room section and suprasternal fossa section. Then, the interatrial septum situation was observed and cardiac abnormalities were excluded.

### Statistical method

Statistical analysis was performed using SPSS 16.0 software (SPSS Inc., Chicago, IL, USA). Measurement data are presented as the mean ± standard deviation. One-factor analysis of variance was used for the mean values of multiple groups and the LSD method was used for pairwise comparison. Linear regression analysis was used to analyze the correlations between FO size, AO size and gestational age. An independent-sample t-test was used for the comparisons between the diagnosis of puerperal ASD (pASD) in the DGWs and the puerperal re-examination of normal FO size and the FO/AO ratio. ROC curve and area under the curve were adopted for the estimation of the demarcation point, sensitivity and specificity of ASD predicted by FO size and FO/AO.

## Results

### General clinical data

The patients were aged 17–45 years with an average age of 28.45±4.34 years. The gestational age was 18–40 weeks with an average of 26.25±3.71 weeks.

Among the 1,690 cases, 958 (56.69%) cases were selected to complete follow-up data, while the remainder (732 cases) were excluded due to fetal cardiac abnormality, imbalance of dextrocardia and levocardia or incomplete relative data.

### Correlation between FO size, AO size and gestational age

**Figure 1:**
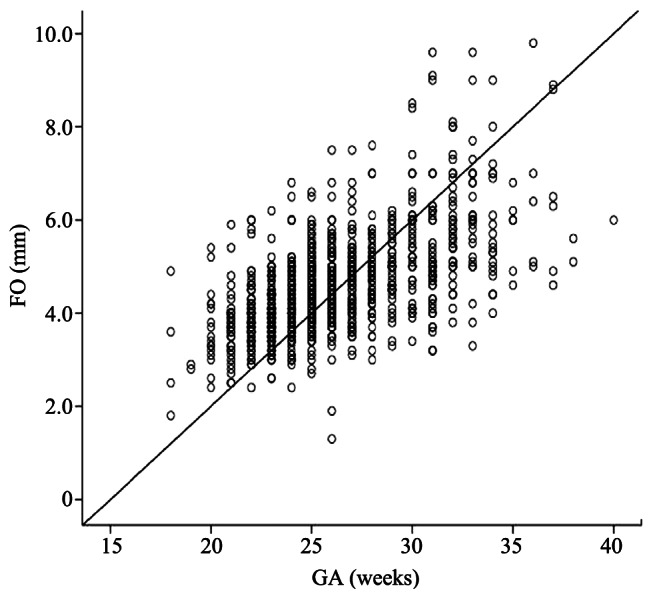
Correlation between fetal FO size and GA in 958 patients at GA 18–40 weeks. The regression equation of fetal FO size and GA is y=0.007+0.175x, P=0.000. FO, foremen ovale; GA, gestational age.

**Figure 2:**
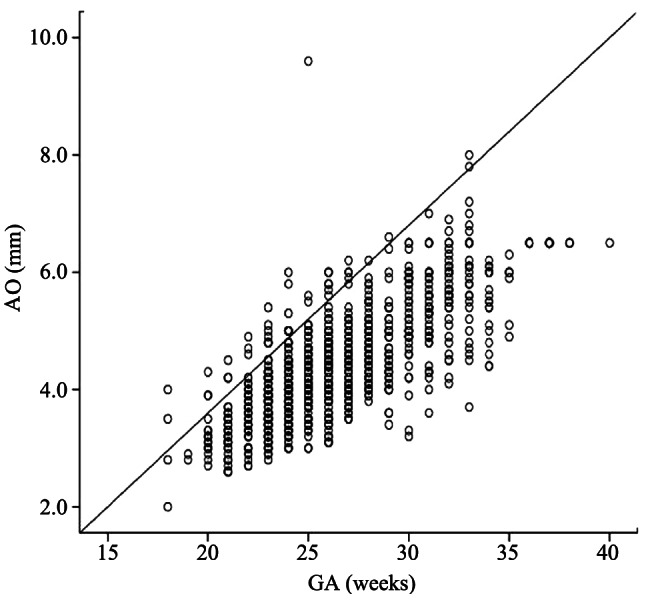
Correlation between fetal FO size and GA in 958 patients at GA 18–40 weeks. The regression equation of fetal FO size and GA is y=0.603 + 0.190x, P=0.000. AO, aorta; GA, gestational age.

Table I presents the measured values of AFO size, AO size and FO/AO in 958 cases from 18 to 40 weeks. The size of the FO at 18–40 weeks increased along with the increasing gestational age (Fig. 1), as well as AO (Fig. 2). The sizes of the FO and AO of all groups were significantly different (P=0.000), while the differences in FO/AO were not significant (P>0.05).

### Comparisons of pASD in DGWs with puerperal normal FO size and FO/AO

**Figure 3:**
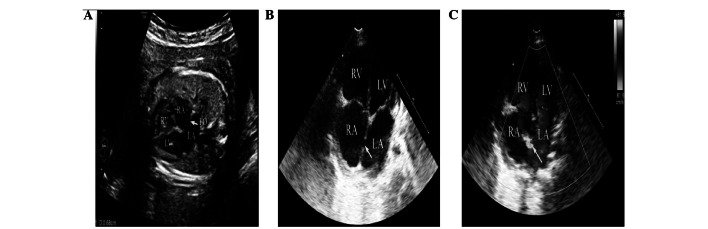
Comparison of antepartum foramen ovale (FO) size and puerperal atrial septal defect. (A) The diameter of the FO was 6.8 mm at 24 weeks; (B) two dimensional echocardiography revealed an atrial septal defect at 12 months after birth; (C) color Doppler flow imaging revealed a left to right atrial shunt. RV, right ventricle; RA, right atrium; LV, left ventricle; LA, left atrium.

Among the 958 cases, there were 31 cases of pASD from 18 to 40 weeks. The measured values of pASD, normal antepartum FO size and FO/AO of the heart are presented in Tables II and III, respectively. These demonstrated significant differences (P=0.000). The size of the puerperal FO at 12 months, re-examined using ultrasonic cardiography, resulted in a diagnosis of ASD in certain patients (Fig. 3).

### Prediction of pASD by ROC curve analysis

Related indicatrix of the ROC curve of pASD predicted by FO size and FO/AO in DGWs are presented in Table IV and V. The areas under the curve of pASD predicted by FO size at 18–22, 23–26, 27–30, 31–34 and 35–40 weeks were 0.991, 0.986, 0.991, 0.998 and 1.000, with demarcation points as 5.02, 5.15, 6.55, 8.55 and 7.90 mm, respectively. Their specificities were 98.2, 90.5, 97.3, 99.2 and 100%, while the sensitivities were all 100%. The areas under the curve of pASD predicted by FO/AO at 18–22, 23–26, 27–30, 31–34 and 35–40 weeks were 0.958, 0.937, 0.974, 0.967 and 0.961, with demarcation points as 1.28, 1.40, 1.32, 1.33 and 1.22, respectively. The sensitivities were 100, 83.3, 100, 100 and 100% and their specificities were 83.0, 78.4, 92.9, 92.2 and 94.1%, respectively. The above results demonstrate that ROC curve analysis of AFO and FO/AO for prediction of pASD is highly accurate.

## Discussion

At the end of the fourth week of fetal development, a diaphragm-like tissue erupts downward on the centre line of the top of the common atrium, which is the septum primum. This diaphragm grows up in the direction of the endocardial cushion, with the formation of an ostiole called the foramen primum before the cushion merges with the diaphragm. With the coalition of the inferior margin of the septum primum and the endocardial cushion, the foramen primum is sealed off and the heart atrium is divided into the left and right atrium. Then, the upper local tissues are dissolved and absorbed gradually, forming a tunnel gallery called the foramen secundum. Additionally, on the top of the right atrium of the septum primum, a diaphragm-like tissue erupts, which is the septum secundum. With the complete anastomosis of the inferior margin of the septum secundum and the endocardial cushion, the diaphragm spreads downward and covers the upper foramen secundum, behind which there is an ostiole called the FO. The thinner valve-like tissues block the FO from the left atrium, performing a valve-like function on the blood circulation of the fetus. The ‘FO valve’ prevents the blood that has entered the left atrium from the right atrium from reversing. Following birth, the FO valve is pressed toward the septum secundum as the pressure of the left atrium is higher than that of the right, leading to the closure of the FO. If the FO is oversized, ASD of the foramen secundum occurs as the septum primum is not able to provide a shade for the FO.

Fetal FO has a growth tendency, similar to other organs. In 1990, Wilson *et al*(7) determined that the FO of gestational aged fetuses are almost equal size to the aortic root inner diameter, or the d-value was <1.0 mm, by means of observing the FO and AO roots of 48 fetuses. In 1994, Phillipos *et al*(3) demonstrated, by analyzing the correlation between fetal FO size and gestational age (GA) of 100 cases from 20 to 38 gestational weeks, that the fetal FO enlarges with increasing gestational age. By analyzing 958 cases divided into five groups according to the number of gestational weeks, we identified that FO size and AO size among all the groups demonstrated statistical differences, FO/AO did not increase with increasing gestational age and FO/AO presented no significant difference among all the groups. In the present study, we demonstrated that the pASD demarcation point (DP) predicted by the FO size at 18–34 gestational weeks increases with the augmentation of gestational age. However, the DP decreases at 35–40 gestational weeks, which may be related to the gradual closure of the FO in the late stage of pregnancy. A number of scholars have noted that fetal hemodynamics may be altered when the FO size is smaller than normal (8–16). In the current study, we observed the FO of DGWs in detail, by dividing the 958 cases into different groups, which provided referred evidence for certain fetuses accompanied with congenital heart growth restriction or early closure of the FO.

The FO is one of the special channels involved in sustaining normal blood circulation (17–19). As the FO closes gradually after birth, infants aged >1 year generally present echo interruption in the middle of the atrial septum and color Doppler reveals that the atrial level shunts from left to right, which may be diagnosed as ASD. Certain researchers suggested that when the size of the FO in the fetal period is >8.0 mm, it should be considered as ASD, while the puerperal follow-up periods identified that not all FO >8 mm led to ASD. Additionally, certain FO <8 mm resulted in ASD. Thus, it is difficult to predict pASD via the observation of the size of the FO. Theoretically speaking, the size of the FO in DGWs during the fetal period grows with the increase of gestational age. Therefore, the prediction of pASD using only one value is not accurate. In the present study, we divided 958 cases into groups according to the number of gestational weeks. Then, relative referenced data were obtained by utilizing ROC curve analysis for the evaluation of the size of the FO to predict the value of pASD in DGWs and to analyze the correlations among fetal FO size, AO size and FO/AO in DGWs. The false positive rate of ASD when the FO closes naturally within a year of birth may be reduced by rechecking the ultrasound cardiogram of patients when they are aged >12 months. The puerperal diagnosed FO size and AO size, and the rechecked normal FO size and AO size were significantly different, indicating that measured values of FO size and FO/AO of pASD were larger than puerperal re-examined values. The results of pASD predicted by FO size and FO/AO in DGWs revealed that the size of the FO in DGWs produced different DPs, specifically, the DPs of pASD predicted by FO size also increased with increasing gestational age, which contributed to the growth tendency of the FO; the 35–40 week gestational age was an exception, when the DPs decreased instead. This study demonstrated that FO/AO is not correlated with gestational age. The DP of pASD predicted by FO/AO was 1.22–1.40. Therefore, there is clinical value in utilizing the size of the FO combined with FO/AO for the prediction of ASD. In this study, the area under the ROC curve, for the prediction of pASD via AFO size and FO/AO in DGWs, was >0.9, illustrating the high accuracy of diagnosis with this method.

In conclusion, the AFO size and FO/AO of pASD in DGWs were greater than those of a normal heart. Prediction of pASD was more accurate when combining the above two indices (AFO size and FO/AO in DGWs). Due to the lack of sample capacity in this study, an increase in sample numbers is required for a more indepth investigation.

## Figures and Tables

**Table I:** Comparisons between antepartum FO size, AO size and FO/AO in DGWs.

Gestational age (weeks)	Number of cases	FO (mm)	AO (mm)	FO/AO
18–22	118	3.82±0.79^a^	3.42±0.50^a^	1.13±0.22
23–26	455	4.27±0.77^a^	4.06±0.64^a^	1.06±0.22
27–30	232	4.90±0.93^a^	4.71±0.70^a^	1.05±0.21
31–34	133	5.70±1.31^a^	5.57±0.78^a^	1.04±0.26
35–40	20	6.22±1.47^a^	6.26±0.47^a^	0.99±0.23

Measurement data are presented as mean ± standard deviation.

a Differences among groups were statistically significant (P=0.000). DGWs, different gestational weeks; FO, foramen ovale; AO, aorta; FO/AO, ratio of the FO size to the AO size.

**Table II:** Comparison of pASD in DGWs with puerperal re-examined normal antepartum and puerperal FO sizes.

	pASD		Puerperal normal	
Gestational age (weeks)	Number of cases	Antepartum FO (mm)	Number of cases	Antepartum FO (mm)
18–22	5	5.76±0.23^a^	113	3.73±0.69
23–26	12	6.23±0.62^a^	443	4.22±0.70
27–30	6	7.53±0.79^a^	226	4.83±0.82
31–34	5	9.26±0.31^a^	128	5.56±1.13
35–40	3	9.17±0.55^a^	17	5.71±0.78

Measurement data are presented as mean ± standard deviation.

a Comparisons between the puerperal diagnosed ASD in DGWs and the puerperal re-examined normal antepartum and puerperal FO revealed significant differences (P=0.000). pASD, puerperal atrial septal defect; DGWs, different gestational weeks; FO, foramen ovale.

**Table III:** Comparisons of pASD in DGWs with puerperal re-examined normal antepartum and puerperal FO/AO.

	pASD		Puerperal normal	
Gestational age (weeks)	Number of cases	Antepartum FO/AO (mm)	Number of cases	Antepartum FO/AO
18–22	5	1.62±0.27^a^	113	1.10±0.19
23–26	12	1.50±0.21^a^	443	1.05±0.20
27–30	6	1.54±0.18^a^	226	1.04±0.19
31–34	5	1.60±0.28^a^	128	1.01±0.23
35–40	3	1.41±0.87^a^	17	0.92±0.16

Measurement data are presented as mean ± standard deviation.

a Comparisons between the puerperal reexamined ASD in DGWs and the puerperal re-examined normal antepartum and puerperal FO/AO were significantly different (P=0.000). pASD, puerperal atrial septal defect; FO/AO, the ratio of the foramen ovale (FO) size to the aorta (AO) size; DGWs, different gestational weeks.

**Table IV:** Relative indices of the ROC curve for prediction of pASD by FO size at different gestational ages.

GA (weeks)	Area under curve	95% confidence interval	DP (mm)	Sensitivity (%)	Specificity (%)
18–22	0.991	0.975–1.000	5.02	100	98.2
23–26	0.986	0.972–1.000	5.15	100	90.5
27–30	0.991	0.979–1.000	6.55	100	97.3
31–34	0.998	0.994–1.000	8.55	100	99.2
35–40	1.000	1.000–1.000	7.90	100	100

GA, gestational age; DP, demarcation point; pASD, puerperal atrial septal defect; FO, foramen ovale.

**Table V:** Relative indexes of ROC curve for prediction of pASD by FO/AO at different gestational ages.

GA (weeks)	Area under curve	95% confidence interval	DP (mm)	Sensitivity (%)	Specificity (%)
18–22	0.958	0.898–1.000	1.28	100	83.0
23–26	0.937	0.879–0.994	1.40	83.3	78.4
27–30	0.974	0.947–1.000	1.32	100	92.9
31–34	0.967	0.933–1.000	1.33	100	92.2
35–40	0.961	0.874–1.000	1.22	100	94.1

GA, gestational age; DP, demarcation point; FO/AO, ratio of the size of the foramen ovale to the size of the aorta; pASD, puerperal atrial septal defect.
